# Prediction of a Cephalometric Parameter and Skeletal Patterns from Lateral Profile Photographs: A Retrospective Comparative Analysis of Regression Convolutional Neural Networks

**DOI:** 10.3390/jcm13216346

**Published:** 2024-10-23

**Authors:** Shota Ito, Yuichi Mine, Shiho Urabe, Yuki Yoshimi, Shota Okazaki, Mizuho Sano, Yuma Koizumi, Tzu-Yu Peng, Naoya Kakimoto, Takeshi Murayama, Kotaro Tanimoto

**Affiliations:** 1 Department of Orthodontics and Craniofacial Developmental Biology, Graduate School of Biomedical and Health Sciences, Hiroshima University, Hiroshima 734-8553, Japan; shota0313@hiroshima-u.ac.jp (S.I.); yukimihsoy@hiroshima-u.ac.jp (Y.Y.); ykoizumi@hiroshima-u.ac.jp (Y.K.); tkotaro@hiroshima-u.ac.jp (K.T.); 2 Department of Medical Systems Engineering, Graduate School of Biomedical and Health Sciences, Hiroshima University, Hiroshima 734-8553, Japansoka0320@hiroshima-u.ac.jp (S.O.); m236608@hiroshima-u.ac.jp (M.S.); 3 Project Research Center for Integrating Digital Dentistry, Hiroshima University, Hiroshima 734-8553, Japan; 4 School of Dentistry, College of Oral Medicine, Taipei Medical University, Taipei 11031, Taiwan; typeng@tmu.edu.tw; 5 Department of Oral and Maxillofacial Radiology, Graduate School of Biomedical and Health Sciences, Hiroshima University, Hiroshima 734-8553, Japan; kakimoto-n@hiroshima-u.ac.jp

**Keywords:** artificial intelligence, deep learning, neural network models, cephalometry, early diagnosis

## Abstract

**Background/Objectives**: Cephalometric analysis has a pivotal role in the quantification of the craniofacial skeletal complex, facilitating the diagnosis and management of dental malocclusions and underlying skeletal discrepancies. This study aimed to develop a deep learning system that predicts a cephalometric skeletal parameter directly from lateral profile photographs, potentially serving as a preliminary resource to motivate patients towards orthodontic treatment. **Methods**: ANB angle values and corresponding lateral profile photographs were obtained from the medical records of 1600 subjects (1039 female and 561 male, age range 3 years 8 months to 69 years 1 month). The lateral profile photographs were randomly divided into a training dataset (1250 images) and a test dataset (350 images). Seven regression convolutional neural network (CNN) models were trained on the lateral profile photographs and measured ANB angles. The performance of the models was assessed using the coefficient of determination (*R*^2^) and mean absolute error (MAE). **Results**: The *R*^2^ values of the seven CNN models ranged from 0.69 to 0.73, and the MAE values ranged from 1.46 to 1.53. Among the seven models, InceptionResNetV2 showed the highest success rate for predictions of ANB angle within 1° of range and the highest performance in skeletal class prediction, with macro-averaged accuracy, precision, recall, and F1 scores of 73.1%, 78.5%, 71.1%, and 73.0%, respectively. **Conclusions**: The proposed deep CNN models demonstrated the ability to predict a cephalometric skeletal parameter directly from lateral profile photographs, with 71% of predictions being within 2° of accuracy. This level of accuracy suggests potential clinical utility, particularly as a non-invasive preliminary screening tool. The system’s ability to provide reasonably accurate predictions without radiation exposure could be especially beneficial for initial patient assessments and may enhance efficiency in orthodontic workflows.

## 1. Introduction

Orthodontists perform a range of clinical assessments on patients to ascertain the etiology of dental malocclusions, develop a treatment plan, and evaluate treatment outcomes. The presence of skeletal malocclusions in adult orthodontic treatment increases the difficulty of treatment and may affect post-treatment stability, and cephalometric analysis is used as a diagnostic basis for malocclusions [1]. Cephalometric radiography is an angle- and distance-normalized radiographic technique used on the skull and is a pivotal modality for the quantification of the craniofacial skeletal complex, facilitating the diagnosis and management of dental malocclusions and underlying skeletal discrepancies [2]. Furthermore, the superimposition of serial cephalometric radiographs allows for prognostication regarding craniofacial growth and for monitoring of treatment efficacy [3]. Orthodontic diagnosis and treatment planning rely extensively on cephalometric analysis, which requires specialized training and expertise for it to be interpreted and applied effectively, which may limit its accessibility in certain regions or healthcare settings [4]. It is important to follow proper radiation safety protocols to minimize the risk of radiation exposure to patients. In particular, radiographic imaging for pediatric patients is intended to be used only after sufficient clinical examinations have been completed [5,6].

The field of Artificial Intelligence (AI) is rapidly advancing, and its applications in oral healthcare are becoming increasingly prevalent [7]. AI applications may benefit from advanced technology and enhanced data analytics, incorporating a broad range of data from multiple sources, such as data at the individual level, environmental level, and system level, to better understand how these factors interact and provide patients with more personalized dental care [8]. Machine learning (ML), a subset of AI, focuses on developing algorithms that enable computers to learn and improve from experience without being explicitly programmed. Deep learning (DL) is a subfield of ML that utilizes artificial neural networks with multiple layers to automatically learn hierarchical features from raw input data. Among the various AI algorithms proposed, convolutional neural network (CNN)-based DL methods have exhibited exceptional performance in the analysis of medical images owing to their superior object recognition abilities [7,9]. The ability of CNNs to automatically learn and recognize complex patterns such as anatomical structures makes them particularly suitable for cephalometric analysis and other medical image processing tasks. The convolutional layers in CNNs are designed to detect local features and spatial hierarchies in images, crucial for identifying and analyzing the intricate details of craniofacial structures in lateral profile photographs and cephalograms. CNNs are designed to process data with a grid-like topology, such as images, by applying a series of convolutional, pooling, and fully connected layers. The convolutional layers detect local features from the input image using a set of learnable filters. The pooling layers downsample the feature maps, reducing their spatial dimensions while retaining the most important information. The fully connected layers then combine the extracted features to make predictions or classifications. Through this hierarchical learning process, CNNs can automatically learn to recognize complex patterns and features in images without the need for manual feature engineering [10]. AI research pertaining to orthodontics has gained significant momentum, including studies on automated cephalometric landmark annotation [4,11], diagnosis of mandibular lateral deviation [12], and prognostication in patients with Class III malocclusion [13]. In particular, automated cephalometric approaches have had some success in streamlining work and reducing inter-operator variability compared to manual landmark annotation in traditional cephalometric analysis [4,11]. These AI systems have the potential to standardize access to orthodontic diagnostics, particularly benefiting resource-limited regions. By enabling remote, automated preliminary assessments, they could significantly enhance the reach and efficiency of orthodontic care delivery, addressing global disparities in access to specialized dental services.

Sagittal skeletal patterns are assessed on the basis of the anterior–posterior position of the maxilla and mandible, which can cause convex-type profiles and maxillary protrusion in the case of a maxilla-forward position, and concave-type profiles and mandibular protrusion in the case of a mandible-forward position [14]. The most commonly used parameter to evaluate sagittal skeletal patterns is the ANB angle, which is determined by the relationship between the anterior limit of the maxillary and mandibular dental bases and the nasion [15]. An excessive ANB angle indicates maxillary protrusion, while a diminished ANB angle suggests mandibular protrusion.

Herein, we propose a DL system that predicts the ANB angle and skeletal patterns from lateral profile photographs without using cephalometric radiographs. Lateral profile photographs are considered suitable for orthodontic diagnostic screening by AI because they can be easily obtained by patients themselves. In the first part of this study, we evaluated the performance of seven CNN models in prediction of the ANB angle from lateral profile photographs. Then, in the second part of this study, we analyzed skeletal patterns based on the ANB angles predicted by this CNN analysis.

## 2. Materials and Methods

### 2.1. Study Design

This retrospective study was approved by the Ethical Committee for Epidemiology of Hiroshima University (Approval Number: E2020-2119) in accordance with the Helsinki Declaration. The requirement for informed consent was waived by the Ethical Committee, with subjects having the opportunity to opt out. All facial photographs were anonymized using a numerical identification system. These identifiers were linked only to skeletal parameters, age, and sex, ensuring that no personally identifiable information remained in the dataset used for analysis.

The reference standard ANB angle values and corresponding lateral profile photographs were obtained from 1600 medical records. The reference standard ANB angle values were determined by orthodontists, each with over eight years of experience affiliated with our institution, using lateral cephalograms obtained during routine practice. The reference standard ANB angle values were determined by orthodontists affiliated with our institution using lateral cephalograms obtained during routine practice. The distribution of the subjects by age is provided in Table 1. The subjects were aged from 3 years 8 months to 69 years 1 month (1039 female and 561 male). Individuals presenting with congenital pathologies impacting occlusal and skeletal configuration, as well as those undergoing or having undergone orthodontic intervention, were excluded from the study. The list of congenital diseases excluded from the current study is in Table S1. The lateral cephalograms on which the ANB angles were measured were recorded in DICOM format using a cephalometric scanner (CX-150W; Asahi Roentgen Ind. Co., Ltd., Kyoto, Japan) between May 2014 and January 2022. All lateral cephalograms were exported as JPEG images. The image resolution of the lateral cephalograms was 1648 × 1980 pixels. The ANB angle values and corresponding lateral profile photographs were used as the dataset. The cephalograms were classified into three groups according to the ANB angle: skeletal Class I with an ANB angle of 1–5°; skeletal Class II with an ANB angle > 5°; and skeletal Class III with an ANB angle < 1° [14].

Because of the availability of 1600 lateral cephalograms and corresponding lateral profile photographs collected during routine care, no formal sample size estimation was performed [16].

### 2.2. CNN Models

To predict the ANB angles from lateral profile photographs, we adapted the following seven CNN models: VGG16 [17], VGG19 [17], InceptionV3 [18], Inception-ResNetV2 [19], DenseNet-121 [20], EfficientNetB7 [21], and EfficientNetV2 [22]. VGG16 and VGG19, developed by the Visual Geometry Group at the University of Oxford, are characterized by their simplicity and depth, consisting of 16 and 19 convolutional layers, respectively [17]. InceptionV3 and Inception-ResNetV2 are based on the Inception architecture, which introduces factorized convolutions and residual connections to improve computational efficiency and training speed [18,19]. DenseNet-121, a variant of the Densely Connected Convolutional Networks, leverages dense connectivity patterns where each layer receives inputs from all preceding layers, enhancing feature propagation and reducing the vanishing gradient problem [20]. EfficientNetB7 and EfficientNetV2 are members of the EfficientNet family, which achieves good performance by systematically scaling the network depth, width, and resolution [21,22]. A transfer learning approach pre-trained with ImageNet was employed for these models. Because the image dimensions of the lateral profile photographs taken with a digital camera were different, the images were cropped using Python algorithms to a square that included point A, point B, the nasal point, anatomical porion, Frankfurt plane, and submandibular plane.

**Figure 1:**
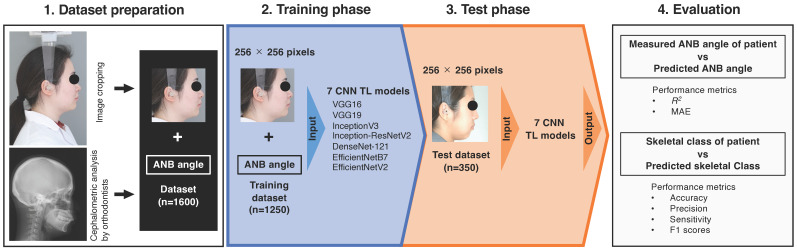
Schematic overview of the prediction of ANB angle and skeletal class with regression CNNs. CNN, convolutional neural network; TL, transfer learning; *R*^2^, coefficient of determination; MAE, mean absolute error.

A schematic of the study is provided in Figure 1. The cropped lateral profile photographs were randomly divided by the Python program into approximately 80% (1250 images) of the training dataset and 20% (350 images) of the test dataset. These images were resized to 256 × 256 pixels with a Python program (ver. 3.8.10) for input into the CNN models. The optimizer used Stochastic Gradient Descent for VGG16 and VGG19, and RMSprop for the remaining five models. The CNN models were trained for a maximum of 100 epochs, and the initial learning rate was set to 1.0 × 10^−5^ for all models. If the loss function did not change for 5 consecutive epochs, the learning rate was reduced by a factor of 0.5, and if the loss function did not change for 20 consecutive epochs, the training was terminated by an early stopping program. All procedures were performed using an NVIDIA GeForce RTX 3090 24 GB graphics card (NVIDIA, Santa Clara, CA, USA). All CNNs were developed using Python and Keras (ver. 2.4.3) with TensorFlow (ver. 2.5.0) as the backend.

### 2.3. Performance Metrics

To evaluate the performance of the CNN models, the coefficient of determination (*R*^2^) and mean absolute error (MAE) were calculated for the 350 test images. The MAE measures the average absolute difference between the predicted and actual values, providing an intuitive interpretation of the model’s performance. The MAE was calculated using the following formula:

(1) $$MAE=\frac{1}{n}\sum_{i=1}^{n}\left|y_{i}-{y^{\prime }}_{i}\right|$$

where *n* is the number of samples, *yi* is the measured ANB angle of subjects analyzed by orthodontists using lateral cephalograms, and *yi*′ is the ANB angle predicted from lateral profile photographs. In the context of ANB angle prediction, an MAE of 1 would indicate that, on average, the predicted angles deviate from the actual angles by 1°.

To further assess the clinical applicability of the models, we calculated the success prediction rates for various precision ranges. The success prediction rate represents the percentage of predictions that fall within a specified range of the actual ANB angles. For each precision range (≤1°, ≤2°, ≤3°, ≤4°, and ≤5°), we calculated the absolute difference between the predicted and actual ANB angles for each sample in the test dataset. The success prediction rate for a given precision range was then computed as the number of samples with absolute differences within that range divided by the total number of samples, expressed as a percentage.

In order to ascertain the reliability and stability of the models, we employed a bootstrap method to compute the 95% confidence intervals (CIs) for both *R*^2^ and MAE metrics.

The performance of the seven regression CNNs for skeletal class prediction was assessed in terms of accuracy, precision, recall, and F1 score [23].

## 3. Results

**Figure 2:**
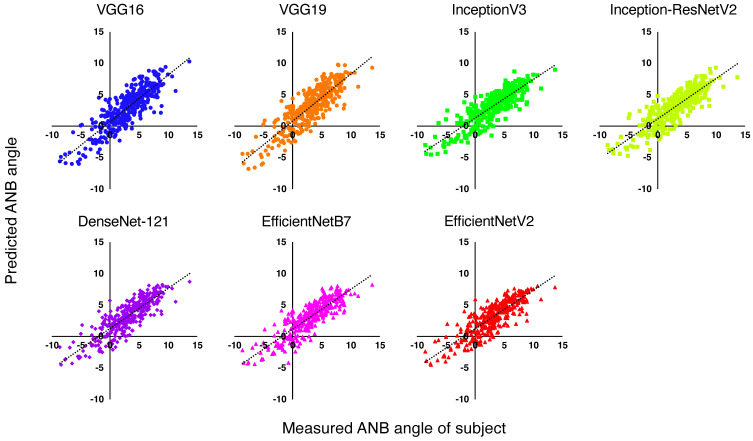
Scatterplot of ANB angles measured from subjects in the test dataset versus the ANB angle predicted by the seven regression CNNs VGG16, VGG19, InceptionV3, Inception-ResNetV2, DenseNet-121, EfficientNetB7, and EfficientNetV2.

Regression analyses using the CNNs were conducted. The predicted outcomes are shown plotted against the true values in Figure 2. The performance values of the various models in predicting the ANB angle are presented in Table 2. The *R*^2^ values of the seven CNN models ranged from 0.69 to 0.73. The MAE values of the seven models ranged from 1.46 to 1.53. The InceptionResNetV2 model exhibited a lower MAE of 1.46 (95% CI, 1.34–1.58) and superior performance compared to the other models. The VGG19 model exhibited a higher *R*^2^ of 0.73 (95% CI, 0.67–0.77) compared to the other models.

**Figure 3:**
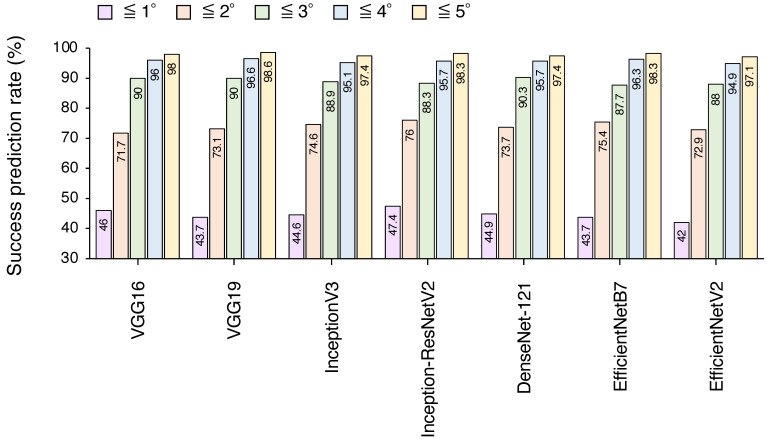
Success prediction rates of the seven regression CNNs for 1°, 2°, 3°, 4°, and 5° precision ranges for the ANB angle values assessed in this study.

The rates of successful predictions of the seven CNN models are shown in Figure 3. The Inception-ResNetV2 model exhibited the highest success rate, with 47.4% of its predictions falling within a 1° range of the actual ANB angles. Moreover, 98.3% of its predictions were within a 5° range, indicating its potential for clinical screening purposes. The success prediction rates for the other models are also shown in Figure 3, demonstrating their performance across different precision ranges.

**Figure 4:**
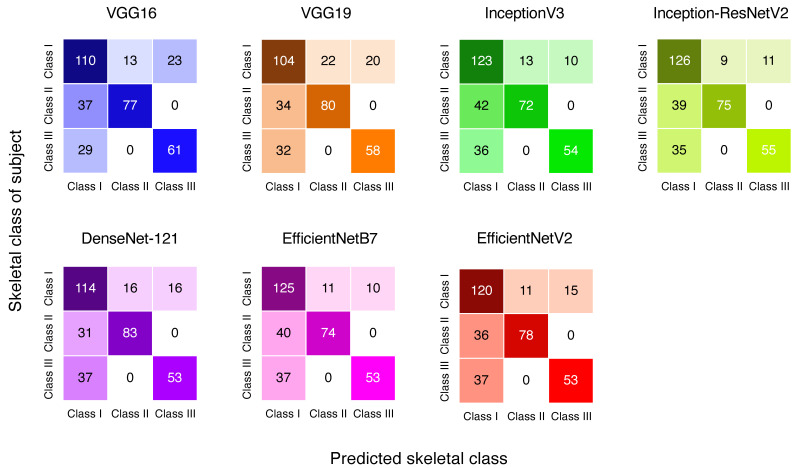
Confusion matrices for the predictions of skeletal class made by the seven regression CNNs.

The skeletal pattern prediction results are presented in Figure 4 in the form of a confusion matrix. The performance metrics are shown in Table 3, along with the macro average scores for skeletal pattern prediction results that indicate the overall performance across classes. InceptionResNetV2 showed the highest performance metrics for the skeletal class prediction, with macro-averaged accuracy, precision, recall, and F1 scores of 73.1%, 78.5%, 71.1%, and 73.0%, respectively. Considering the multiple performance metrics above, InceptionResNetV2 provided the most accurate results in this study.

## 4. Discussion

In the medical field, a variety of imaging techniques are used to acquire patient-specific data [24]. Hence, the use of CNNs for diagnostic support and image analysis has received considerable research attention, and a number of CNN-based techniques have already been integrated into clinical practice [8,25]. Recently, regression CNNs have been utilized for the quantitative estimation of clinical parameters from radiographic images [26]. Regression CNNs are used as a method of training a neural network to perform linear regression on the input data, rather than simply classifying the data into predefined categories [27]. Making use of this technology, we attempted to use a CNN model to predict cephalometric measurements through regression analysis. We found that seven CNN models, VGG16, VGG19, InceptionV3, Inception-ResNetV2, DenseNet-121, EfficientNetB7, and EfficientNetV2, could predict the ANB angle from lateral profile photographs, and of these seven, the VGG19 model exhibited a higher *R*^2^ of 0.73 (95% CI, 0.67–0.77) compared to the other models. The InceptionResNetV2 model exhibited a lower MAE of 1.46 (95% CI, 1.34–1.58), showing better results than the other models. In assessing the successful prediction rate of the ANB angle, the InceptionResNetV2 model exhibited superior performance, with 47.4% of discrepancies between the measured and CNN-predicted ANB angle contained within 1° of range. InceptionResNetV2 also showed the highest performance in the skeletal class prediction, with the highest macro-averaged accuracy, precision, recall, and F1 scores. Although VGG19 and EfficientNetB7 also showed good values for MAE and *R*^2^, InceptionResNetV2 was considered to have the best results when all performance metrics were considered. In our study, we used the ANB angle as one of the criteria for skeletal class classification, with 1–5° considered as the standard range. Previous studies have suggested that errors within 2 mm or 2° are generally tolerable and do not significantly impact clinical decision-making [28,29]. Our model’s MAE of 1.46° demonstrates good performance. Moreover, errors exceeding 4° could potentially lead to misclassification of skeletal class, which would be clinically critical. To understand the distribution of these errors, we evaluated deviations of ≤1° to ≤5°, in addition to MAE and *R*^2^. An analysis of the confusion matrices revealed consistent patterns of misclassification across all models. Errors were observed in distinguishing between Classes I and III, as well as between Classes I and II. Notably, no model made the clinically critical error of misclassifying Class II as Class III or vice versa. This finding suggests that while our models may struggle with borderline cases, they successfully avoid the most serious misclassifications that could lead to inappropriate treatment planning.

We were unable to find other studies using CNN models to predict cephalometric parameters from directly input lateral profile photographs, making comparisons with similar studies difficult. While numerous AI algorithms and CNN models have been proposed, selecting the optimal model requires careful consideration of the intended use and the size of the dataset [30,31]. Theoretical prediction of the most suitable network architecture is a challenging task, necessitating a reliance on trial-and-error experimentation, as well as practical experience. Ali et al. trained an artificial neural network (ANN) on the relationship between skeletal measurements obtained from lateral cephalograms and measurements obtained from lateral profile photographs annotated with anatomical landmarks on soft tissue [32]. Through this approach, the ANN was able to accurately predict the skeletal measurements of lateral cephalograms using skeletal measurements obtained from lateral profile photographs. However, although this method avoids the radiation exposure associated with lateral cephalometry, it still requires the time-consuming task of annotating anatomical landmarks and measuring skeletal parameters on lateral profile photographs, similar to the process of lateral cephalometric analysis. We consider our method using a CNN to be a more convenient and promising approach because lateral profile photographs are directly input into the models and the models predict the skeletal measurement values that would be obtained from cephalograms without subjecting the patient to radiation exposure. Our dataset included patients with mandibular protrusion or maxillary protrusion due to significant skeletal malocclusion and mandibular deviation caused by posterior cross-bite that required early treatment intervention or further growth evaluation.

It is postulated that pediatric patients would benefit significantly from AI-assisted diagnosis that eliminates the need for radiation exposure. This approach could be extended to both pediatric and adult patients, allowing them to perform initial AI-based screenings using lateral profile images taken with their mobile devices. Such accessibility may encourage timely visits to orthodontists. In addition, this technology has potential applications in telemedicine, which could be particularly beneficial in resource-limited regions. In areas lacking cephalometric analysis tools, the ability to conduct initial orthodontic screenings remotely using only lateral profile photographs could significantly expand access to care. This method could also be valuable for monitoring treatment progress, assessing patient compliance, and detecting early signs of recurrence. The implementation of AI-assisted analysis of lateral profile photographs not only reduces radiation exposure, but also provides a convenient and accessible tool for preliminary orthodontic assessments. This approach is in line with the growing trend of digital health solutions and has the potential to improve the efficiency and reach of orthodontic care.

The present study has several limitations. First, the lateral profile photographs forming the dataset contained multiple sources of information, such as soft tissue and hair, which may influence the predictions. Individuals sharing the same ANB angle can nonetheless present distinct soft tissue profiles. Influential factors, such as retrusion and protrusion of lateral incisors, can significantly contribute to these variations. To enhance the performance of the predictions, supplementary data such as sex and/or gender, race, weight, body fat percentage, and body mass index may be necessary. Second, while we constructed a CNN-based system that predicts the ANB angle from the input of lateral profile photographs, the accuracy of skeletal pattern classification based on the predicted ANB angle is currently approximately 70%, which limits its usefulness for orthodontic diagnosis. The ANB angle is measured based on the nasion and is a cephalometric analysis item that evaluates the anteroposterior relationship of the maxilla and mandible [15], but it alone cannot accurately classify the skeletal pattern. In clinical practice, various parameters from the Downs analysis, such as the Angle of Convexity, Facial Angle, A-B Plane angle [33], and Wits appraisal [34], are used to evaluate the anteroposterior relationship of the jaws comprehensively. Our study does not address vertical skeletal patterns evaluated using the Frankfort mandibular angle [33,35], Sella–Nasion-to-mandibular-plane angle [36], etc., which are essential for creating a clinically useful AI model. Future research will involve training on multiple cephalometric parameters and weighting them to achieve more accurate skeletal pattern classification. Third, this study used a relatively restricted dataset obtained from a single institution. The patients excluded from this study had a rare disease known to cause disease-specific malformations in the craniomaxillofacial region. Rare diseases were excluded to ensure the homogeneity of the dataset, as they are associated with atypical skeletal and facial features, as well as a history of corrective surgery, and it is difficult to collect adequate training data for each disease [37]. For patients with a history of orthodontic intervention, we recognized the potential for orthodontic appliances or orthognathic surgery to modify the lateral profile. To maintain the integrity of this study, we systematically excluded data from patients who had undergone orthodontic treatment at the time of their inaugural visit to our hospital, as well as those currently in treatment, thereby eliminating any appliance-induced or artificial alterations in the lateral profile. Ensuring secure data handling will be critical when considering the possibility of patients using mobile devices for initial screening. Future implementation of this technology will require robust privacy protocols and clear communication with patients about the uses and limitations of AI-based diagnostics. Ensuring reliable and equitable performance of these models in diverse populations is critical for ethical and effective clinical application. These considerations will be essential for future research as we work towards the development of comprehensive and ethically sound AI-based diagnostic systems.

## 5. Conclusions

This research successfully created a deep learning-based system for predicting cephalometric skeletal parameters from lateral profile photographs. The deep CNNs proposed in this study demonstrated promising results, with 71% of test images assessed within 2° of accuracy. This level of accuracy potentially offers a valuable preliminary tool for patient motivation towards orthodontic treatment, serving as a pre-stage to initial examinations including cephalometric analysis. The system’s ability to provide reasonably accurate predictions from lateral profile photographs could streamline the initial assessment process, potentially enhancing patient engagement and treatment planning efficiency.

## Figures and Tables

**Table 1:** The distribution of the subjects by age.

Age (Year)		0–5		6–10		11–20		21–30		31–40		41–50		51–60		61–70		Total
Sex		F	M	F	M	F	M	F	M	F	M	F	M	F	M	F	M	
Skeletal Class	I	1	1	163	132	169	106	76	18	21	3	12	2	5	4	0	1	714
	II	3	1	89	76	110	46	65	14	26	2	27	3	6	1	3	0	472
	III	2	2	62	39	124	69	48	30	13	6	6	3	8	0	0	2	414
Total		6	4	314	247	403	221	189	62	60	11	45	8	19	5	3	3	1600

**Table 2:** Performance of the seven regression CNNs for ANB prediction.

CNN Model	*R^2^*	95% CI	MAE	95% CI
VGG16	0.72	0.65–0.77	1.50	1.37–1.63
VGG19	0.73	0.67–0.77	1.50	1.38–1.62
InceptionV3	0.71	0.66–0.75	1.49	1.37–1.62
Inception-ResNetV2	0.72	0.68–0.77	1.46	1.34–1.58
DenseNet-121	0.71	0.66–0.75	1.50	1.38–1.62
EfficientNetB7	0.72	0.67–0.76	1.48	1.36–1.60
EfficientNetV2	0.69	0.64–0.74	1.53	1.40–1.67

**Table 3:** Performance metrics of the seven regression CNNs for skeletal class prediction.

CNN Model	Skeletal Class	Precision (%)	Recall (%)	F1 Score (%)	Accuracy (%)
VGG16	Class I	62.5	75.3	68.3	−
	Class II	85.6	67.5	75.5	−
	Class III	72.6	67.8	70.1	−
	Macro average	73.6	70.2	71.3	70.9
VGG19	Class I	61.2	71.2	65.8	−
	Class II	78.4	70.2	74.1	−
	Class III	74.4	64.4	69.0	−
	Macro average	71.3	68.6	69.6	69.1
InceptionV3	Class I	61.2	84.2	70.9	−
	Class II	70.6	63.2	66.7	−
	Class III	69.2	60.0	64.3	−
	Macro average	67.0	69.1	67.3	71.1
Inception-ResNetV2	Class I	63	86.3	72.8	−
	Class II	89.3	65.8	75.8	−
	Class III	83.3	61.1	70.5	−
	Macro average	78.5	71.1	73.0	73.1
DenseNet-121	Class I	62.6	78.1	69.5	−
	Class II	83.0	72.8	77.6	−
	Class III	77.9	58.9	67.1	−
	Macro average	74.5	70.0	71.4	71.4
EfficientNetB7	Class I	63.1	85.6	72.7	−
	Class II	87.1	64.9	74.4	−
	Class III	79.1	58.9	67.5	−
	Macro average	76.4	69.8	71.5	72.0
EfficientNetV2	Class I	64.8	82.2	72.5	−
	Class II	86.7	68.4	76.5	−
	Class III	70.7	58.9	64.2	−
	Macro average	74.1	69.8	71.1	71.7

## Data Availability

The data presented in this study are available on request from the corresponding author.

## Supplementary Materials

The following supporting information can be downloaded at: https://www.mdpi.com/article/10.3390/jcm13216346/s1, Table S1: The list of congenital diseases excluded from the current study.
