# Supplementary material for: Prediction of a Cephalometric Parameter and Skeletal Patterns from Lateral Profile Photographs: A Retrospective Comparative Analysis of Regression Convolutional Neural Networks
Source: J Clin Med. 2024 Oct 23;13(21):6346. doi: 10.3390/jcm13216346 (PMC11546473; doi:10.3390/jcm13216346)
Supplement: Supplementary file 1 [file jcm-13-06346-s001.zip › jcm-3271931-supplementary.pdf]

Supplementary Materials

Table S1. The list of congenital diseases excluded from the current study

CHARGE syndrome
Williams syndrome
Klippel-Trenaunay-Weber syndrome
Cleft lip and/or palate
Goldenhar syndrome
Syndromic Craniosynostosis
Sturge-Weber syndrome
Stickler syndrome
Fibrous dysplasia
Sotos syndrome
Turner syndrome
Down syndrome
Treacher-Collins syndrome
Noonan syndrome
Pierre Robin sequence
Prader Willi Syndrome
Beckwith-Wiedemann syndrome
Marfan syndrome
Moebius syndrome
Larsen syndrome
Lymphangioma
Rubinstein-Taybi syndrome
Romberg syndrome
Kabuki syndrome
Gorlin syndrome
Muscular Dystrophy
Osteogenesis imperfecta
Cleidocranial dysplasia
Chromosomal deletion syndrome
von Recklinghausen disease

Growth hormone deficiency dwarfism

Osteopetrosis

Achondroplasia
